# Rare germline copy number variants (CNVs) and breast cancer risk

**DOI:** 10.1038/s42003-021-02990-6

**Published:** 2022-01-18

**Authors:** Joe Dennis, Jonathan P. Tyrer, Logan C. Walker, Kyriaki Michailidou, Leila Dorling, Manjeet K. Bolla, Qin Wang, Thomas U. Ahearn, Irene L. Andrulis, Hoda Anton-Culver, Natalia N. Antonenkova, Volker Arndt, Kristan J. Aronson, Laura E. Beane Freeman, Matthias W. Beckmann, Sabine Behrens, Javier Benitez, Marina Bermisheva, Natalia V. Bogdanova, Stig E. Bojesen, Hermann Brenner, Jose E. Castelao, Jenny Chang-Claude, Georgia Chenevix-Trench, Christine L. Clarke, NBCS Collaborators, Vessela N. Kristensen, Kristine K. Sahlberg, Anne-Lise Børresen-Dale, Inger Torhild Gram, Olav Engebråten, Bjørn Naume, Jürgen Geisler, Grethe I. Grenaker Alnæs, J. Margriet Collée, CTS Consortium, James Lacey, Elena Martinez, Fergus J. Couch, Angela Cox, Simon S. Cross, Kamila Czene, Peter Devilee, Thilo Dörk, Laure Dossus, A. Heather Eliassen, Mikael Eriksson, D. Gareth Evans, Peter A. Fasching, Jonine Figueroa, Olivia Fletcher, Henrik Flyger, Lin Fritschi, Marike Gabrielson, Manuela Gago-Dominguez, Montserrat García-Closas, Graham G. Giles, Anna González-Neira, Pascal Guénel, Eric Hahnen, Christopher A. Haiman, Per Hall, Antoinette Hollestelle, Reiner Hoppe, John L. Hopper, Anthony Howell, ABCTB Investigators, Christine Clarke, Jane Carpenter, Deborah Marsh, Rodney Scott, Robert Baxter, Desmond Yip, Alison Davis, Nirmala Pathmanathan, Peter Simpson, Dinny Graham, Mythily Sachchithananthan, kConFab/AOCS Investigators, Ian Campbell, Anna de Fazio, Stephen Fox, Judy Kirk, Geoff Lindeman, Roger Milne, Melissa Southey, Amanda Spurdle, Heather Thorne, Agnes Jager, Anna Jakubowska, Esther M. John, Nichola Johnson, Michael E. Jones, Audrey Jung, Rudolf Kaaks, Renske Keeman, Elza Khusnutdinova, Cari M. Kitahara, Yon-Dschun Ko, Veli-Matti Kosma, Stella Koutros, Peter Kraft, Vessela N. Kristensen, Katerina Kubelka-Sabit, Allison W. Kurian, James V. Lacey, Diether Lambrechts, Nicole L. Larson, Martha Linet, Alicja Ogrodniczak, Arto Mannermaa, Siranoush Manoukian, Sara Margolin, Dimitrios Mavroudis, Roger L. Milne, Taru A. Muranen, Rachel A. Murphy, Heli Nevanlinna, Janet E. Olson, Håkan Olsson, Tjoung-Won Park-Simon, Charles M. Perou, Paolo Peterlongo, Dijana Plaseska-Karanfilska, Katri Pylkäs, Gad Rennert, Emmanouil Saloustros, Dale P. Sandler, Elinor J. Sawyer, Marjanka K. Schmidt, Rita K. Schmutzler, Rana Shibli, Ann Smeets, Penny Soucy, Melissa C. Southey, Anthony J. Swerdlow, Rulla M. Tamimi, Jack A. Taylor, Lauren R. Teras, Mary Beth Terry, Ian Tomlinson, Melissa A. Troester, Thérèse Truong, Celine M. Vachon, Camilla Wendt, Robert Winqvist, Alicja Wolk, Xiaohong R. Yang, Wei Zheng, Argyrios Ziogas, Jacques Simard, Alison M. Dunning, Paul D. P. Pharoah, Douglas F. Easton

**Affiliations:** 1 Centre for Cancer Genetic Epidemiology, Department of Public Health and Primary Care, University of Cambridge, Cambridge, UK; 2 Centre for Cancer Genetic Epidemiology, Department of Oncology, University of Cambridge, Cambridge, UK; 3 Department of Pathology and Biomedical Science, University of Otago, Christchurch, New Zealand; 4 Biostatistics Unit, The Cyprus Institute of Neurology & Genetics, Nicosia, Cyprus; 5 Cyprus School of Molecular Medicine, The Cyprus Institute of Neurology & Genetics, Nicosia, Cyprus; 6 Division of Cancer Epidemiology and Genetics, National Cancer Institute, National Institutes of Health, Department of Health and Human Services, Bethesda, MD USA; 7 Fred A. Litwin Center for Cancer Genetics, Lunenfeld-Tanenbaum Research Institute of Mount Sinai Hospital, Toronto, ON Canada; 8 Department of Molecular Genetics, University of Toronto, Toronto, ON Canada; 9 Department of Medicine, Genetic Epidemiology Research Institute, University of California Irvine, Irvine, CA USA; 10 N.N. Alexandrov Research Institute of Oncology and Medical Radiology, Minsk, Belarus; 11 Division of Clinical Epidemiology and Aging Research, German Cancer Research Center (DKFZ), Heidelberg, Germany; 12 Department of Public Health Sciences, and Cancer Research Institute, Queen’s University, Kingston, ON Canada; 13 Department of Gynecology and Obstetrics, Comprehensive Cancer Center Erlangen-EMN, University Hospital Erlangen, Friedrich-Alexander University Erlangen-Nuremberg (FAU), Erlangen, Germany; 14 Division of Cancer Epidemiology, German Cancer Research Center (DKFZ), Heidelberg, Germany; 15 Biomedical Network on Rare Diseases (CIBERER), Madrid, Spain; 16 Human Cancer Genetics Programme, Spanish National Cancer Research Centre (CNIO), Madrid, Spain; 17 Institute of Biochemistry and Genetics, Ufa Federal Research Centre of the Russian Academy of Sciences, Ufa, Russia; 18 Department of Radiation Oncology, Hannover Medical School, Hannover, Germany; 19 Gynaecology Research Unit, Hannover Medical School, Hannover, Germany; 20 Copenhagen General Population Study, Herlev and Gentofte Hospital, Copenhagen University Hospital, Herlev, Denmark; 21 Department of Clinical Biochemistry, Herlev and Gentofte Hospital, Copenhagen University Hospital, Herlev, Denmark; 22 Faculty of Health and Medical Sciences, University of Copenhagen, Copenhagen, Denmark; 23 Division of Preventive Oncology, German Cancer Research Center (DKFZ) and National Center for Tumor Diseases (NCT), Heidelberg, Germany; 24 German Cancer Consortium (DKTK), German Cancer Research Center (DKFZ), Heidelberg, Germany; 25 Oncology and Genetics Unit, Instituto de Investigacion Sanitaria Galicia Sur (IISGS), Xerencia de Xestion Integrada de Vigo-SERGAS, Vigo, Spain; 26 Cancer Epidemiology Group, University Cancer Center Hamburg (UCCH), University Medical Center Hamburg-Eppendorf, Hamburg, Germany; 27 Department of Genetics and Computational Biology, QIMR Berghofer Medical Research Institute, Brisbane, QLD Australia; 28 Westmead Institute for Medical Research, University of Sydney, Sydney, NSW Australia; 29 Department of Clinical Genetics, Erasmus University Medical Center, Rotterdam, The Netherlands; 30 Department of Laboratory Medicine and Pathology, Mayo Clinic, Rochester, MN USA; 31 Sheffield Institute for Nucleic Acids (SInFoNiA), Department of Oncology and Metabolism, University of Sheffield, Sheffield, UK; 32 Academic Unit of Pathology, Department of Neuroscience, University of Sheffield, Sheffield, UK; 33 Department of Medical Epidemiology and Biostatistics, Karolinska Institutet, Stockholm, Sweden; 34 Department of Pathology, Leiden University Medical Center, Leiden, The Netherlands; 35 Department of Human Genetics, Leiden University Medical Center, Leiden, The Netherlands; 36 Nutrition and Metabolism Section, International Agency for Research on Cancer (IARC-WHO), Lyon, France; 37 Channing Division of Network Medicine, Department of Medicine, Brigham and Women’s Hospital and Harvard Medical School, Boston, MA USA; 38 Department of Epidemiology, Harvard T.H. Chan School of Public Health, Boston, MA USA; 39 Division of Evolution and Genomic Sciences, School of Biological Sciences, Faculty of Biology, Medicine and Health, University of Manchester, Manchester Academic Health Science Centre, Manchester, UK; 40 North West Genomics Laboratory Hub, Manchester Centre for Genomic Medicine, St Mary’s Hospital, Manchester University NHS Foundation Trust, Manchester Academic Health Science Centre, Manchester, UK; 41 David Geffen School of Medicine, Department of Medicine Division of Hematology and Oncology, University of California at Los Angeles, Los Angeles, CA USA; 42 Usher Institute of Population Health Sciences and Informatics, The University of Edinburgh, Edinburgh, UK; 43 Cancer Research UK Edinburgh Centre, The University of Edinburgh, Edinburgh, UK; 44 The Breast Cancer Now Toby Robins Research Centre, The Institute of Cancer Research, London, UK; 45 Department of Breast Surgery, Herlev and Gentofte Hospital, Copenhagen University Hospital, Herlev, Denmark; 46 School of Public Health, Curtin University, Perth, WA Australia; 47 Fundación Pública Galega de Medicina Xenómica, Instituto de Investigación Sanitaria de Santiago de Compostela (IDIS), Complejo Hospitalario Universitario de Santiago, SERGAS, Santiago de Compostela, Spain; 48 Moores Cancer Center, University of California San Diego, La Jolla, CA USA; 49 Cancer Epidemiology Division, Cancer Council Victoria, Melbourne, Victoria, Australia; 50 Centre for Epidemiology and Biostatistics, Melbourne School of Population and Global Health, The University of Melbourne, Melbourne, VIC Australia; 51 Precision Medicine, School of Clinical Sciences at Monash Health, Monash University, Clayton, VIC Australia; 52 Center for Research in Epidemiology and Population Health (CESP), Team Exposome and Heredity, INSERM, University Paris-Saclay, Villejuif, France; 53 Center for Familial Breast and Ovarian Cancer, Faculty of Medicine and University Hospital Cologne, University of Cologne, Cologne, Germany; 54 Center for Integrated Oncology (CIO), Faculty of Medicine and University Hospital Cologne, University of Cologne, Cologne, Germany; 55 Department of Preventive Medicine, Keck School of Medicine, University of Southern California, Los Angeles, CA USA; 56 Department of Oncology, Södersjukhuset, Stockholm, Sweden; 57 Department of Medical Oncology, Erasmus MC Cancer Institute, Rotterdam, The Netherlands; 58 Dr. Margarete Fischer-Bosch-Institute of Clinical Pharmacology, Stuttgart, Germany; 59 University of Tübingen, Tübingen, Germany; 60 Division of Cancer Sciences, University of Manchester, Manchester, UK; 61 Department of Genetics and Pathology, Pomeranian Medical University, Szczecin, Poland; 62 Independent Laboratory of Molecular Biology and Genetic Diagnostics, Pomeranian Medical University, Szczecin, Poland; 63 Department of Epidemiology & Population Health, Stanford University School of Medicine, Stanford, CA USA; 64 Department of Medicine, Division of Oncology, Stanford Cancer Institute, Stanford University School of Medicine, Stanford, CA USA; 65 Division of Genetics and Epidemiology, The Institute of Cancer Research, London, UK; 66 Division of Molecular Pathology, The Netherlands Cancer Institute - Antoni van Leeuwenhoek Hospital, Amsterdam, The Netherlands; 67 Department of Genetics and Fundamental Medicine, Bashkir State University, Ufa, Russia; 68 Radiation Epidemiology Branch, Division of Cancer Epidemiology and Genetics, National Cancer Institute, Bethesda, MD USA; 69 Department of Internal Medicine, Evangelische Kliniken Bonn gGmbH, Johanniter Krankenhaus, Bonn, Germany; 70 Translational Cancer Research Area, University of Eastern Finland, Kuopio, Finland; 71 Institute of Clinical Medicine, Pathology and Forensic Medicine, University of Eastern Finland, Kuopio, Finland; 72 Biobank of Eastern Finland, Kuopio University Hospital, Kuopio, Finland; 73 Program in Genetic Epidemiology and Statistical Genetics, Harvard T.H. Chan School of Public Health, Boston, MA USA; 74 Department of Medical Genetics, Oslo University Hospital and University of Oslo, Oslo, Norway; 75 Institute of Clinical Medicine, Faculty of Medicine, University of Oslo, Oslo, Norway; 76 Department of Histopathology and Cytology, Clinical Hospital Acibadem Sistina, Skopje, Republic of North Macedonia; 77 Department of Computational and Quantitative Medicine, City of Hope, Duarte, CA USA; 78 City of Hope Comprehensive Cancer Center, City of Hope, Duarte, CA USA; 79 VIB Center for Cancer Biology, Leuven, Belgium; 80 Laboratory for Translational Genetics, Department of Human Genetics, University of Leuven, Leuven, Belgium; 81 Department of Health Sciences Research, Mayo Clinic, Rochester, MN USA; 82 Unit of Medical Genetics, Department of Medical Oncology and Hematology, Fondazione IRCCS Istituto Nazionale dei Tumori di Milano, Milan, Italy; 83 Department of Clinical Science and Education, Södersjukhuset, Karolinska Institutet, Stockholm, Sweden; 84 Department of Medical Oncology, University Hospital of Heraklion, Heraklion, Greece; 85 Department of Obstetrics and Gynecology, Helsinki University Hospital, University of Helsinki, Helsinki, Finland; 86 School of Population and Public Health, University of British Columbia, Vancouver, BC Canada; 87 Cancer Control Research, BC Cancer, Vancouver, Canada; 88 Department of Cancer Epidemiology, Clinical Sciences, Lund University, Lund, Sweden; 89 Department of Genetics, Lineberger Comprehensive Cancer Center, University of North Carolina at Chapel Hill, Chapel Hill, NC USA; 90 Genome Diagnostics Program, IFOM - the FIRC Institute of Molecular Oncology, Milan, Italy; 91 Research Centre for Genetic Engineering and Biotechnology ‘Georgi D. Efremov’, MASA, Skopje, Republic of North Macedonia; 92 Laboratory of Cancer Genetics and Tumor Biology, Cancer and Translational Medicine Research Unit, Biocenter Oulu, University of Oulu, Oulu, Finland; 93 Laboratory of Cancer Genetics and Tumor Biology, Northern Finland Laboratory Centre Oulu, Oulu, Finland; 94 Clalit National Cancer Control Center, Carmel Medical Center and Technion Faculty of Medicine, Haifa, Israel; 95 Department of Oncology, University Hospital of Larissa, Larissa, Greece; 96 Epidemiology Branch, National Institute of Environmental Health Sciences, NIH, Research Triangle Park, NC USA; 97 School of Cancer & Pharmaceutical Sciences, Comprehensive Cancer Centre, Guy’s Campus, King’s College London, London, UK; 98 Division of Psychosocial Research and Epidemiology, The Netherlands Cancer Institute - Antoni van Leeuwenhoek hospital, Amsterdam, The Netherlands; 99 Center for Molecular Medicine Cologne (CMMC), Faculty of Medicine and University Hospital Cologne, University of Cologne, Cologne, Germany; 100 Department of Surgical Oncology, University Hospitals Leuven, Leuven, Belgium; 101 Genomics Center, Centre Hospitalier Universitaire de Québec – Université Laval Research Center, Québec City, QC Canada; 102 Department of Clinical Pathology, The University of Melbourne, Melbourne, Victoria, Australia; 103 Division of Breast Cancer Research, The Institute of Cancer Research, London, UK; 104 Department of Population Health Sciences, Weill Cornell Medicine, New York, NY USA; 105 Epigenetic and Stem Cell Biology Laboratory, National Institute of Environmental Health Sciences, NIH, Research Triangle Park, NC USA; 106 Department of Population Science, American Cancer Society, Atlanta, GA USA; 107 Department of Epidemiology, Mailman School of Public Health, Columbia University, New York, NY USA; 108 Department of Epidemiology, Gillings School of Global Public Health and UNC Lineberger Comprehensive Cancer Center, University of North Carolina at Chapel Hill, Chapel Hill, NC USA; 109 Department of Quantitative Health Sciences, Division of Epidemiology, Mayo Clinic, Rochester, MN USA; 110 Institute of Environmental Medicine, Karolinska Institutet, Stockholm, Sweden; 111 Department of Surgical Sciences, Uppsala University, Uppsala, Sweden; 112 Division of Epidemiology, Department of Medicine, Vanderbilt Epidemiology Center, Vanderbilt-Ingram Cancer Center, Vanderbilt University School of Medicine, Nashville, TN USA; 113 Department of Research, Vestre Viken Hospital, Drammen, Norway; 114 Department of Tumor Biology, Institute for Cancer Research, Oslo University Hospital-Radiumhospitalet, Oslo, Norway; 115 Department of Cancer Genetics, Institute for Cancer Research, Oslo University Hospital-Radiumhospitalet, Oslo, Norway; 116 Department of Community Medicine, The Arctic University of Norway, Tromsø, Norway; 117 Department of Oncology, Division of Surgery and Cancer and Transplantation Medicine, Oslo University Hospital-Radiumhospitalet, Oslo, Norway; 118 Department of Oncology, Akershus University Hospital, Lørenskog, Norway; 119 University of California, San Diego, CA USA; 120 The Westmead Institute for Medical Research, The University of Sydney, Sydney, NSW Australia; 121 University of Technology Sydney, Translational Oncology Group, School of Life Sciences, Faculty of Science, Ultimo, NSW Australia; 122 School of Biomedical Sciences, University of Newcastle, Newcastle; Hunter Medical Research Institute and NSW Health Pathology North, Newcastle, Australia; 123 Kolling Institute of Medical Research, University of Sydney, St Leonards, NSW Australia; 124 Epigenetics & Transcription Laboratory Melanie Swan Memorial Translational Centre, Sci-Tech, University of Canberra, Canberra, Australia; 125 Department of Medical Oncology, The Canberra Hospital, Garran, ACT Australia; 126 The Canberra Hospital, Garran, ACT Australia; 127 The Australian National University, Canberra, ACT Australia; 128 Westmead Breast Cancer Institute, Western Sydney Local Health District, Westmead, NSW Australia; 129 University of Sydney, Western Clinical School, Westmead, NSW Australia; 130 UQ Centre for Clinical Research, Faculty of Medicine, The University of Queensland, Herston, QLD Australia; 131 Peter MacCallum Cancer Centre, Melbourne, Australia; 132 Westmead Institute for Cancer Research, Sydney, Australia; 133 Department of Medicine, Westmead Hospital, Sydney, Australia; 134 Walter and Eliza Hall Institute, Melbourne, Australia; 135 Queensland Institute of Medical Research, Brisbane, Australia

**Keywords:** Breast cancer, Genome-wide association studies

## Abstract

Germline copy number variants (CNVs) are pervasive in the human genome but potential disease associations with rare CNVs have not been comprehensively assessed in large datasets. We analysed rare CNVs in genes and non-coding regions for 86,788 breast cancer cases and 76,122 controls of European ancestry with genome-wide array data. Gene burden tests detected the strongest association for deletions in *BRCA1* (*P* = 3.7E−18). Nine other genes were associated with a *p*-value < 0.01 including known susceptibility genes *CHEK2* (*P* = 0.0008), *ATM* (*P* = 0.002) and *BRCA2* (*P* = 0.008). Outside the known genes we detected associations with *p*-values < 0.001 for either overall or subtype-specific breast cancer at nine deletion regions and four duplication regions. Three of the deletion regions were in established common susceptibility loci. To the best of our knowledge, this is the first genome-wide analysis of rare CNVs in a large breast cancer case-control dataset. We detected associations with exonic deletions in established breast cancer susceptibility genes. We also detected suggestive associations with non-coding CNVs in known and novel loci with large effects sizes. Larger sample sizes will be required to reach robust levels of statistical significance.

## Abstract (web summary)

Dennis et al. investigate potential breast cancer associations with rare germline copy number variants (CNVs) by conducting a genome-wide analysis in a large breast cancer case-control dataset. The authors detected associations with exonic deletions in established breast cancer susceptibility genes and suggestive associations for a number of non-coding CNVs.

## Introduction

Copy number variants (CNVs) are pervasive in the human genome but are more challenging to detect with current technologies than single nucleotide variants (SNVs). Recent comprehensive sequencing projects^1,2^ have characterised CNVs in large sample sets. The gnomAD project identified a median of 3,505 deletions and 723 duplications covering more than 50 base pairs per genome. Most deletions and duplications tend to be rare with longer variants tending to be rarer, suggesting negative selection against these variants. At the population level the 1000 Genomes project has mapped a large proportion of inherited CNVs^3^ and observed that 65% had a frequency below 2%.

While somatic copy number alterations play a major role in the pathogenesis of breast tumours^4,5^, some germline CNVs are known to be associated with inherited risk of breast cancer. Rare loss of function variants in susceptibility genes such as *BRCA1* and *CHEK2* are associated with a large increase in risk^6^. While the majority of these variants are single nucleotide mutations and short indels, they also include longer deletions and duplications. It has been reported that up to a third of loss of function *BRCA1* variants in some populations may be CNVs^7^.

Large-scale genome-wide association studies (GWAS) have established breast cancer associations with common variants at more than 150 loci, mostly in non-coding regions^8–11^. At two of the loci, deletions imputed from the 1000 Genomes reference panel have been identified as likely causal variants. A deletion of the *APOBEC3B* gene-coding region increases breast cancer risk^12^ and analysis of the tumours of the germline deletion carriers showed an increase in APOBEC-mediated somatic mutations^13^. A deletion in a regulatory region was identified as a likely causal variant at the 2q35 locus^14,15^.

**Fig. 1:**
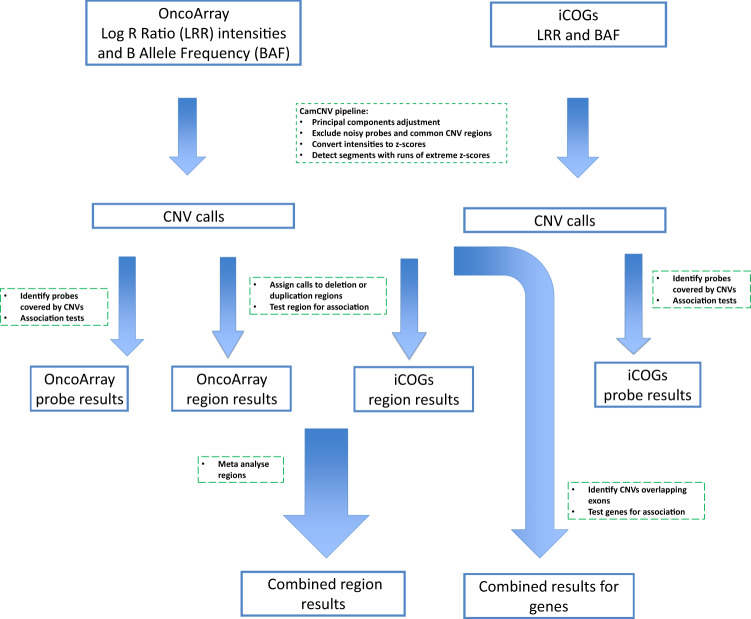
Flow diagram of  the calling and analysis pipeline. CNVs were called using the CamCNV pipeline from the intensities from the iCOGs and OncoArray genotyping arrays. The CNVs were assigned to regions, association results generated for each array and then meta-analysed. CNVs covering the coding regions of genes were analysed in gene burden tests.

Detecting CNVs from the intensity measurements of genotyping array probes is prone to producing unreliable calls due to the high level of noise. We recently developed a new CNV calling method, CamCNV^16^, which focuses on rare CNVs and identifies outlier samples that may have a CNV, based on the intensity distribution across all samples at each probe. We showed that this approach is able to detect CNVs using as few as three probes^16^. Here, we apply this approach to a very large array genotype dataset to search for breast cancer-associated CNVs. The analyses are outlined in Fig. 1.

## Results

### Summary of CNVs detected

After quality control we detected a mean of 2.9 deletions (standard deviation 1.6) and 2.5 duplications (SD 2.0) per sample. Supplementary Data 5 shows the mean length, probe coverage, and segment z-scores of called CNVs. Duplications tended to be longer than deletions: for example, deletions called on OncoArray covered a mean of 45 Kilobases (Kb) (SD 106 Kb) over 9.8 probes (SD 17.2), while duplications covered a mean of 109 Kb (SD 202 Kb) over 18.9 probes (SD 36.5). CNV calls observed in multiple samples were concentrated in a small proportion of probes (Supplementary Data 6), with <11% of probes having frequency >0.01% and <2% of probes having frequency >0.5%.

We identified called CNVs which overlapped for at least 90% of their length with rare deletions and duplications (frequency < 1%) identified by the 1000 Genomes Project (Supplementary Data 5 and Supplementary Fig. 1.). Forty-nine percent of OncoArray deletions and 47% of iCOGs deletions matched a 1000 Genomes Project variant while 29% of OncoArray duplications and 20% of iCOGs duplications matched. In total, we identified CNVs closely matching 3273 of the deletion variants published by the 1000 Genomes Project (~9% of total) and 1255 of their duplication variants (~24% of the total).

### CNVs associated with overall risk

**Fig. 2:**
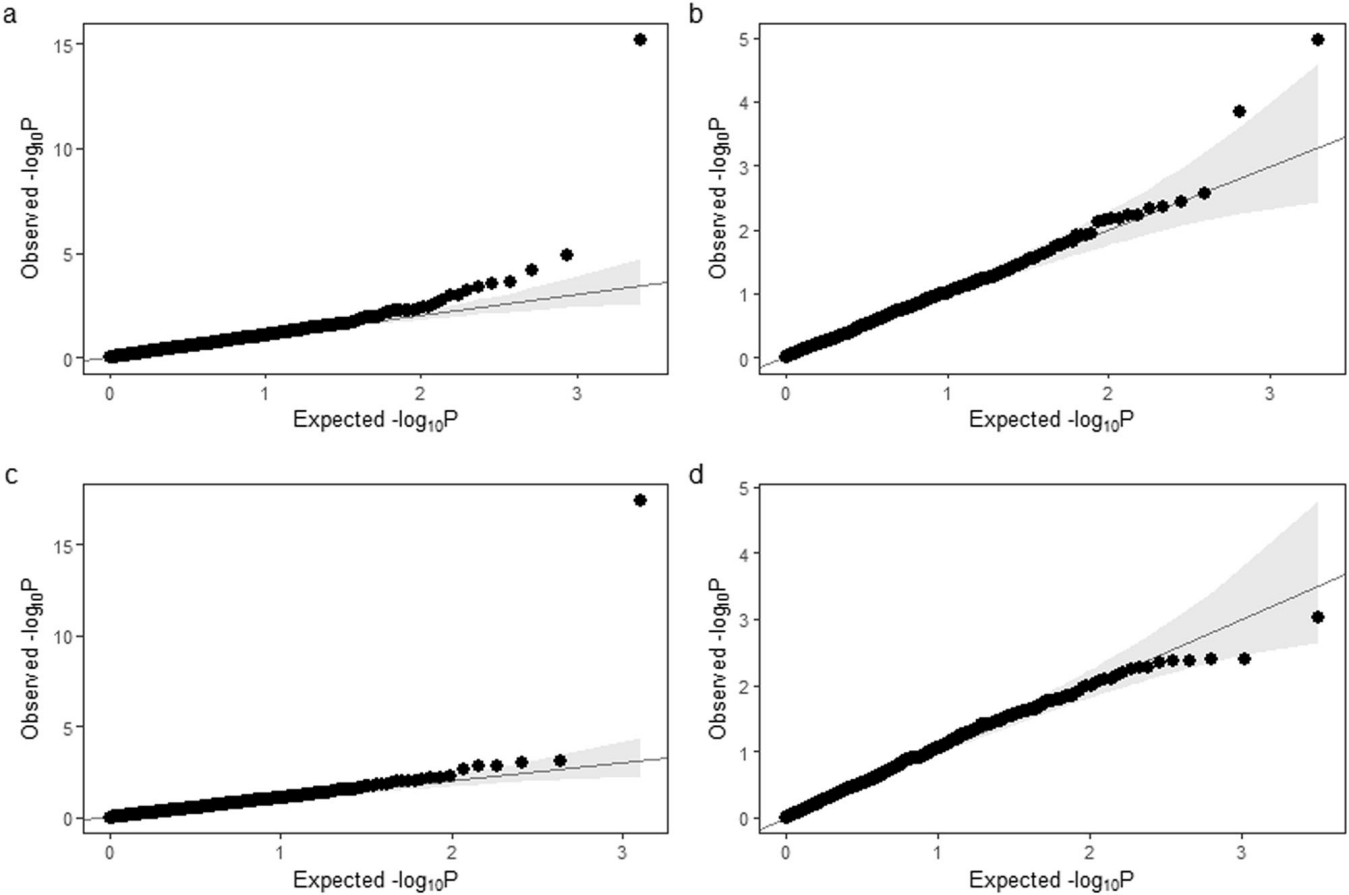
QQ plots of association results. Quantile-quantile plots of *P*-values from association tests of deletion regions (**a**), duplication regions (**b**), gene burden analysis for deletions (**c**), and gene burden for duplications (**d**).

Association results were derived for 1301 regions containing deletions and 992 regions containing duplications. QQ plots are shown in Fig. 2a for deletions and 2b for duplications. There was no evidence for inflation in the test statistics for duplications (lambda = 0.98; lambda_1000_ = 1.00) and minimal evidence for deletions (lambda = 1.11; lambda_1000_ = 1.00).

Seven deletion and two duplication regions were associated with breast cancer risk at *p* < 0.001 (Table 1); of these, deletions within the *BRCA1* region achieved *p* < 10^−6^. The results for all regions are shown in Supplementary Data 7 and 8 and include statistics on the number of probes covered by the calls. The results for individual probes covered by the regions analysed are in Supplementary Data 9−12.

**Table 1:** CNVs associated with overall risk.

Type	Locus	Chr.	Start (Build37)	End	Total carriers	Odds ratio	Lower CI	Upper CI	Direction (OncoArray, iCOGs)	*P*-value
Deletion	BRCA1	17	41188342	41363651	195	6.27	4.02	9.79	++	6.32E−16
Deletion	Intergenic_FGFR2_ATE1	10	123435817	123461066	630	1.42	1.21	1.67	++	1.42E−05
Deletion	Intergenic_ADCY8_EFR3A	8	132199447	132250643	42	4.88	2.24	10.61	++	6.36E−05
Deletion	KLHL1	13	70652321	71029916	1761	0.85	0.77	0.92	−−	2.31E−04
Deletion	CHEK2	22	29083731	29123846	141	1.94	1.35	2.79	++	3.26E−04
Deletion	SUPT3H	6	44908728	45244478	32	0.23	0.1	0.52	−?	4.25E−04
Deletion	Intergenic_GALNT1_C18orf21	18	33350917	33359197	123	1.92	1.32	2.78	?+	6.24E−04
Duplication	17p13.3_VPS53;NXN	17	13905	1559829	577	0.69	0.59	0.82	−−	1.08E−05
Duplication	21q22.11_HUNK_LINC00159	21	33410933	33863246	102	2.23	1.47	3.38	++	1.48E−04

The *BRCA1* locus contains multiple deletions across the gene. The *CHEK2* region (OR 1.94, *p* = 0.0003) covers the whole gene but nearly all the calls correspond to a deletion of exons nine and ten, which was previously observed in 1% of breast cancer cases and 0.4% of controls in Poland^17^. We observed the deletion in 0.9% of Polish cases and 0.5% of controls.

**Fig. 3:**
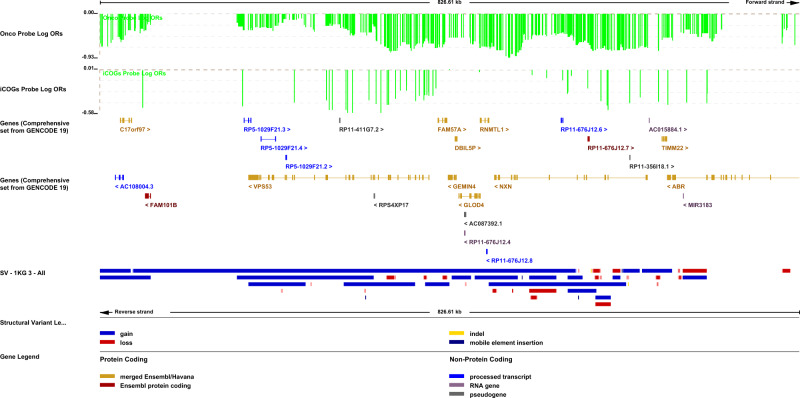
Associations with duplications at the 17p13.3 locus. Log odds ratios for individual probes from Oncoarray and iCOGs are shown at the top in green. Genes from the Ensembl browser are shown in the next row, followed by structural variants identified by the 1000 Genomes Project with duplications in blue and deletions in red.

The most significant association (OR = 0.69 *P* = 0.00001) for duplications covers a large region on 17p13.3 with multiple long variants overlapping shorter duplications. The OncoArray results by probe show the strongest associations at a series of probes (17: 814529–850542) in the first intron of *NXN*, with the lowest *P*-value at 17: 819141 (OR = 0.45, *P* = 0.002). The most significant probe position on iCOGs was also in this region (17:836631, OR = 0.58, *P* = 0.09) (Fig. 3).

### Associations with risk of breast cancer subtypes

We repeated the analyses restricting cases to those with ER-positive, ER-negative, and triple negative disease. Deletions and duplications with *p*-values below 0.001 are shown in Tables 2 and 3 and the results for all regions are in Supplementary Data 13 and 14. An association was observed for *BRCA1* for all subtypes, with the exception of duplications for ER-positive disease. The odds ratio for *BRCA1* deletions was higher for ER-negative disease (OR = 27.03; 95% CI, 15.66−46.67) than ER-positive (OR = 2.81; CI, 1.56 to 5.08; *P* = 8.46E–28 for the difference), while for *CHEK2* the odds ratio was higher for ER-positive disease (OR = 2.32;CI,1.56–3.44) than ER-negative (OR = 1.36; CI,0.66 to 2.82; *P* = 0.11 for the difference), consistent with the known subtype-specific associations for deleterious variants in these genes^18^.

**Table 2:** Subtype associations for deletions.

Subtype	Locus	Chr.	Start (Build37)	End	Total carriers	Odds ratio	Lower CI	Upper CI	Direction (OncoArray, iCOGs)	*P*-value
ER Positive	Intergenic_FGFR2_ATE1	10	123435817	123461066	478	1.52	1.27	1.81	++	5.04E−06
ER Positive	CHEK2	22	29091788	29102967	79	2.36	1.56	3.44	++	3.03E−05
ER Positive	ITGBL1 intronic	13	102122905	102133221	58	3.29	1.83	5.92	+?	7.29E−05
ER Positive	Intergenic_ADCY8_EFR3A	8	132199447	132250643	31	4.95	2.20	11.30	++	1.07E−04
ER Positive	BRCA1	17	41188342	41363651	57	2.81	1.55	5.08	++	6.41E−04
ER Negative	BRCA1	17	41188342	41363651	112	27.03	15.66	46.67	++	2.62E−32
ER Negative	Intergenic:ABCC4_CLDN10	13	95991263	96004144	134	2.16	1.43	3.26	+?	2.46E−04
ER Negative	KLF12 intronic	13	74356683	74357984	93	2.39	1.49	3.82	+?	2.89E−04
ER Negative	Intergenic_DPP10_DDX18	2	118258797	118389164	44	3.34	1.64	6.80	++	8.84E−04
Triple Neg.	BRCA1	17	41188342	41363651	72	40.55	21.70	75.76	++	3.64E−31
Triple Neg.	Intergenic_DPP10_DDX18	2	118258797	118389164	40	6.56	2.83	15.18	++	1.13E−05
Triple Neg.	Intergenic_DPP10_DDX18	2	117973154	118107795	48	4.54	1.88	10.97	++	7.92E−04

**Table 3:** Subtype associations for duplications.

Subtype	Locus	Chr.	Start (Build37)	End	Total carriers	Odds ratio	Lower CI	Upper CI	Direction (OncoArray, iCOGs)	*P*-value
ER Positive	17p13.3_VPS53;NXN	17	13905	1559829	454	0.67	0.55	0.81	−−	4.44E−05
ER Positive	21q22.11_HUNK_LINC00159	21	33410933	33863246	77	2.55	1.6	4.06	++	7.88E−05
ER Positive	15q13	15	28440287	32797352	1250	0.83	0.75	0.93	−−	6.95E−04
ER Negative	BRCA1	17	41188342	41363651	42	5.93	2.31	15.19	+−	2.09E−04
Triple Neg.	BRCA1	17	41188342	41363651	35	10.80	3.33	35.02	+−	7.29E−05
Triple Neg.	Intergenic_TMC3_MEX3B	15	81960409	82104822	231	2.39	1.55	3.71	++	9.25E−05

In total, we observed five deletion and two duplication regions with *p*-values < 0.001 that did not reach *p* < 0.001 in the overall risk analysis. The strongest novel association for ER-positive was for an intronic deletion in *ITGBL1* (OR = 3.3, *P* = 0.00007, *P* for difference by ER-status = 0.18). For ER-negative disease the strongest novel association was with an intergenic deletion between *ABCC4* (*MRP4*) and *CLDN10* (OR = 2.16, *P* = 0.0002, *P* for difference by ER-status = 0.02). Neither of these associations was significant for the other subtype. For triple negative disease, the strongest evidence of association was found for an intergenic duplication between *TMC3* and *MEX3B* (OR = 2.39, *P* = 0.00009) and for two separate deletions upstream of the *DDX18* gene: 2:118258797–118389164 (OR = 6.56, *P* = 0.00001) and 2:117973154–118107795 (OR = 4.54, *P* = 0.0008). The association at these two deletions was driven by the same samples, with 75% of the carriers of the first deletion observed to have the second deletion and normal copy number at the 62 kb gap between the deletions.

### Associations at established common susceptibility Loci

Three of the most significant associations were observed within regions harbouring known breast cancer susceptibility loci for breast cancer. The most significant (OR = 1.42;CI,1.21−1.67; *P* = 0.00015) was upstream of *FGFR2* and consistent with a 28 kb deletion in the 1000 Genomes Project data (chr10:123433204−123461492). Three independent risk signals have been previously identified at this region^19,20^. The effect size for the CNV was larger than those previously reported for these common SNPs (largest OR = 1.27;CI,1.22−1.25). The CNV is in linkage disequilibrium with two of the SNPs identified as likely causally associated variants: rs35054928 (*D*′ = 0.82) and rs2981578 (*D*′ = 0.88). Conditioning on those SNPs reduced somewhat the strength of the association for the deletion (OR = 1.30; CI 1.10−1.53; *P* = 0.002, Supplementary Data 15).

The third strongest signal (OR = 4.9, *P* = 0.00001) in the deletion analysis for overall breast cancer was at 8: 132199447−132252439, 144Kb downstream of *ADCY8*. The strongest GWAS signal in this region lies in an intron of *ADCY8* (lead SNP rs73348588, OR = 1.13, *P* = 8.2e−7)^9^. A 3 kb deletion in intron 4 of *KLF12* was associated with ER-negative disease (OR = 2.4, *P* = 0.0007, P for difference by ER-status=0.01). This is 389 kb distant from common SNPs, located between *KLF12* and *KLF5*, associated with ER-negative disease (rs9573140, OR = 0.94, *P* = 3.62e−9)^21^. The *KLF12* and *ADCY8* deletions are not in strong linkage disequilibrium with the corresponding GWAS signals and conditioning on these SNPs did not alter the strengths of the association for the CNVs (Supplementary Data 15).

### Gene burden tests

We performed gene burden tests based on CNVs that overlapped exons. Analyses were restricted to genes in which at least 24 CNVs were identified, leaving 645 genes with deletions (Supplementary Data 16) and 1596 genes with duplications (Supplementary Data 17). QQ plots are shown in Fig. 2c for deletions and 2d for duplications. The lambda for inflation was 1.18 (; lambda_1000_ = 1.00) for deletions and 1.07 (; lambda_1000_ = 1.00) for duplications.

For deletions, we found 10 genes with *P* < 0.01 (Table 4), the most significant being *BRCA1* (OR = 7.66, *P* = 3.72E−18). Four of these 10 genes (*ATM, BRCA1, BRCA2,* and *CHEK2*) are known breast cancer susceptibility genes^18^. Deletions were also observed in two other known susceptibility genes: *PALB2* (23 cases, 9 controls, OR = 2.02, *P* = 0.09) and *RAD51C* (21 cases, 9 controls, OR = 2.04, *P* = 0.08). The most significant novel association was for *SUPT3H* (OR = 0.27, *P* = 0.0004).

**Table 4:** Gene burden results for deletions.

Gene	Cases	Controls	Odds ratio	Lower CI	Upper CI	*P*-value
BRCA1	171	22	7.66	4.84	12.13	3.72E−18
CHEK2	103	48	1.83	1.29	2.61	7.66E−04
SUPT3H	10	25	0.27	0.13	0.59	9.24E−04
PCDHGB2	25	3	7.03	2.10	23.52	1.55E−03
MEAK7	59	24	2.19	1.34	3.58	1.66E−03
ATM	35	8	3.43	1.56	7.51	2.11E−03
MAD1L1	54	25	2.00	1.23	3.26	5.53E−03
NPHP1	477	351	1.22	1.06	1.41	6.13E−03
ZNF320	29	7	3.28	1.39	7.73	6.63E−03
BRCA2	65	33	1.81	1.17	2.81	7.82E−03

For duplications we observed 15 genes with *P* < 0.01 (Table 5). The most significant association was for *VPS53* (OR = 0.5, *P* = 0.0009). This gene and *ABR* (OR = 0.61, *P* = 0.008) both lie within the region on 17p which had the strongest association in the regional analysis. These associations were driven by duplications in different samples, with only one duplication in one sample overlapping both genes. Duplications were associated with an increased risk for only four of the 15 genes; the most statistically significant was *RSU1* (OR = 3.4, *P* = 0.004). There was also some evidence of association with risk for duplications in *BRCA1* (OR = 1.75, *P* = 0.01). However, analysis restricted to duplications that included exon 12 of *BRCA1* showed clearer evidence of association (34 carriers, OR = 4.7, *P* = 0.0001), consistent with one of the more frequent known *BRCA1* duplications that results in a frameshift^22^.

**Table 5:** Gene burden results for duplications.

Gene	Cases	Controls	Odds ratio	Lower CI	Upper CI	*P*-value
VPS53	40	65	0.50	0.33	0.75	9.46E−04
ATP12A	48	66	0.57	0.39	0.84	3.97E−03
USP18	12	23	0.34	0.17	0.71	4.16E−03
RPS6KA2	10	22	0.32	0.14	0.70	4.20E−03
RSU1	21	8	3.40	1.47	7.84	4.23E−03
AC008132.1	7	17	0.26	0.10	0.66	4.49E−03
PNPLA4	479	346	1.23	1.06	1.42	5.30E−03
NLGN4X	291	320	0.79	0.67	0.93	5.55E−03
ZNF439	31	9	2.98	1.37	6.45	5.72E−03
TRIM6	5	19	0.24	0.09	0.67	6.34E−03
RP11.363G10.2	13	27	0.39	0.20	0.78	7.39E−03
USP31	7	18	0.30	0.12	0.73	8.02E−03
TRDN	15	29	0.42	0.22	0.80	8.26E−03
ABR	51	69	0.61	0.42	0.88	8.88E−03
DNAJC15	109	67	1.52	1.11	2.09	9.29E−03

The gene burden subtype results are shown in Supplementary Data 18 and 19. The strongest associations were observed for *BRCA1* deletions for ER Negative (OR = 33, *P* = 5.5E−35) and Triple Negative (OR = 49, *P* = 7.1E−34) disease, *CHEK2* deletions for ER positive disease (OR = 2.14, *P* = 0.0001) and *ATM* deletions for ER positive disease (OR = 4.85, *P* = 0.0001). No additional genes significant at *P* < 0.0001 were found.

### Direction of effect tests

In the gene burden and individual probe analyses we observed a directional effect, whereby the strongest associations for deletions tended to increase risk and those for duplications tended to be protective. To test whether these associations deviated from what would be expected by chance, we computed ranked summed z-score tests and evaluated the significance of the maximum test statistic by permutation. Results are summarised in Table 6. The statistic for deletion regions was more significant than any of the permuted statistics (*P* = 0.04) but was reduced to *P* = 0.12 after removing the known genes *BRCA1* and *CHEK2*. The significance of the gene burden test for deletions also was reduced from *P* = 0.04 to *P* = 0.2 when the known genes were removed. The statistic for the duplication regions was lower than any of the 50 permutations (*P* = 0.04). The gene burden analysis for duplications was not significant.

**Table 6:** Direction of effect results.

Category	Analysis ustat	Max.ustat of 50 simulations	Min. ustat of 50 simulations	Simulations with larger/smaller ustat	*P*-value
Deletion regions	9.48	6.96	−7.81	0	0.04
Deletion regions minus known^a^	5.9	6.96	−7.81	2	0.12
Duplication regions	−9.2	6.54	−6.99	0	0.04
Gene deletions	9.18	5.64	−9.67	0	0.04
Gene deletions minus known^b^	5.01	5.64	−9.67	4	0.20
Gene duplications	−4.33	5.96	−11.26	33	1.29

^a^BRCA1, CHEK2 regions excluded.

^b^BRCA1, CHEK2, ATM, BRCA2 removed.

## Discussion

We used the largest available breast cancer case-control dataset, comprising more than 86,000 cases and 76,000 controls with array genotyping, to test for associations with rare CNVs. Using the intensities from genotyping arrays to detect CNVs is not ideal due to a high level of noise and uncertainty in the calling, particularly for duplications. However, in tests of known CNVs and replication of calls between duplicate samples, the CamCNV method shows reasonable levels of sensitivity and specificity^16^. The main focus of this analysis was low frequency CNVs (<1% frequency) since higher frequency CNVs can generally be studied through imputation to a reference panel. In the 0.05–1% frequency range, we could detect ~20% of the CNVs identified by the 1000 Genomes project. For some loci, we only had evidence from one array because the probes do not exist to detect the variants on the other array. Thus, while this array-based approach provides power to evaluate the CNVs that can be assayed, much denser arrays or direct sequencing would be required to provide a complete evaluation of the contribution of CNVs.

In support of the reliability of the method, we detected evidence for both deletions and duplications in *BRCA1*, which was stronger for ER-negative disease, and for deletions in *CHEK2*, which were stronger for ER-positive disease. The latter appeared to be driven by a single founder deletion in East European populations. Weaker evidence of association was also observed for deletions in other susceptibility genes (*BRCA2, ATM, PALB2,* and *RAD51C*); the ORs were consistent with those seen for deleterious SNVs and indels^18^. In total, around 0.5% of cases in our analysis had a deletion in one of the known susceptibility genes with the proportion rising to ~1% for cases diagnosed under 50 years of age. The majority of coding deletions are expected to affect only part of the gene, with one study observing that a quarter covered only a single exon^23^. To detect all of these using array data would require at least three probes per exon. The OncoArray has this level of coverage for a few genes, including *BRCA1* and *BRCA2*, but the coverage is lower for most genes and many coding CNVs will have been missed.

A key issue is the appropriate level of statistical significance to apply to these analyses. For the gene burden tests, *P* < 2.5 × 10^−6^, as used in exome-sequencing, seems an appropriate level. It is less clear what is appropriate for non-coding variants. A level of *P* < 5 × 10^−8^ has become standard in GWAS and has been shown to lead to acceptable replication, but this seems over-conservative for CNVs, which are more likely to be deleterious. Consistent with this, for at least two of the ~200 common susceptibility loci, the likely causal variant is a CNV, a higher fraction than expected given the relative frequencies of CNVs and SNPs. Based on frequency analysis of whole-genome sequence data Abel et al.^1^ estimated that rare CNVs are >800 times more likely to be deleterious than rare SNVs and >300 times more likely than rare indels. On the other hand, the significance level for non-coding CNVs should logically be more stringent than for the gene burden tests. Taken together, a significance level of ~10^−6^ seems appropriate, while associations at *P* < 0.001 may be worth following up in future studies. In our analyses, only the association at *BRCA1* (both in the overall and gene burden tests) passes the higher threshold. We also calculated Bayesian False Discovery Probabilities (BFDPs)^24^ (Supplementary Data 20 and 21) for our associations using prior probabilities of 0.001 for regions and 0.002 for genes. Outside the known genes none of the BDFPs gave a probability below 10%, with the lowest BFDP of 0.11 for the deletion in the *FGFR2* locus. For a CNV observed with a frequency of 0.1% (*n* = 91 samples in the OncoArray dataset) we had 40% power to detect an association with an odds ratio of 2 but only 1.5% power to detect an association with an odds ratio of 1.5. An OR of 2, comparable to that seen for deleterious variants in *ATM* and *CHEK2*, may be plausible for rare coding CNVs or non-coding CNVs that have a significant effect on gene expression. Larger sample sizes will clearly be required to evaluate rare CNVs with more modest associations.

In addition to the *BRCA1* and *CHEK2* loci, we found associations in three known susceptibility regions identified through GWAS, harbouring *FGFR2*, *ADCY8*, and *KLF12*. In each case, the variants are rarer than the established associated variants, but confer higher risks. The *ADCY8* and *KLF12* deletions are not in linkage disequilibrium with the associated SNPs. The *FGFR2* deletion is in linkage disequilibrium with two of the likely causal common SNPs although there was still evidence of association with the deletion, albeit weaker, after conditional analysis. * In-silico* and functional analysis clearly demonstrate that FGFR2 is the target of the previously established variants;^19,20^ it will be interesting to establish if the same is true for the CNV.

Excluding loci in known susceptibility regions, the strongest evidence of association was for a 12 kb deletion (13:102121830–102133956) in the second intron of *ITGBL1* (OR = 3.3, *P* = 0.00007 in the ER-positive analysis). This deletes a promoter flanking region (Ensembl ID: ENSR00001563823) and CTCF binding site (Ensembl ID: ENSR00001062398) active in mammary epithelial cells. There is experimental evidence that *ITGBL1* expression, mediated by the RUNX2 transcription factor, enables breast cancer cells to form bone metastases^25^.

In the gene burden analysis, the strongest novel association was for deletions within *SUPT3H*, which were associated with a reduced risk. *SUPT3H* encodes human SPT3, a component of the STAGA complex that acts as a co-activator of the MYC oncoprotein^26^. *SUPTH* is located within the first intron of the *RUNX2* transcription factor and the syntenic relationship between the two genes is highly evolutionarily conserved^27^. *RUNX2* has a role in mammary gland development and high *RUNX2* expression is found in ER-negative tumours^28^. The *PCDHGB2* association appears to be due to a single variant (5:140739812–140740918) that deletes the first exon but as this gene is part of the protocadherin gamma gene cluster it is also possible that the deletion may be having an effect on one of the five genes that overlap *PCDHGB2*. It also deletes a promoter active in mammary epithelial cells (ENSR00001342785). The next strongest signals were for *MEAK7* (OR = 2.19, *P* = 0.001), a gene implicated in a mTOR signalling pathway^29^, and *MAD1L1* ((OR = 2.00, *P* = 0.005), a component of the mitotic spindle-assembly that has been suggested as a possible tumour suppressor^30^.

After *BRCA1*, the most significant association for ER Negative disease in the gene burden analysis was for *CYP2C18* which overlaps *CYP2C19* (ER-negative OR = 2.6, *P* = 0.002; triple-negative OR = 4.4, *P* = 0.0002). A previous analysis of CNVs and breast cancer in the Finnish population identified a founder mutation reaching an overall frequency of ~ 3% and reported a possible association at this locus for triple negative (OR 2.8, *p* = 0.02) and ER-negative breast cancer (OR = 2.2, *p* = 0.048)^31^.

The results from duplications are harder to interpret as there are often longer duplications overlapping whole genes with shorter variants covering some part of their length. For the gene burden analysis there was little evidence of strong associations. In the regional analysis, the two strongest associations cover multiple genes. The strongest evidence of association (OR = 0.69, *P* = 1.1E−05) was for a 1.5 Mb region at the start of chromosome 17 (17:13905–1559829). The probe-specific and gene burden results highlighted some stronger signals within this region, for example within *NXN* and *VPS53*, but the direction of effect was consistent across the region with 80% of the OncoArray probes having an odds ratio of 0.75 or lower (Fig. 3). This locus has established associations with prostate and colorectal cancer. Interestingly a possible association with ER-positive breast cancer survival was detected for a rare SNP in the first intron of NXN, rs118021774 (HR = 1.83, *P* = 3.8E−06)^32^. The detected duplications are not in LD with this SNP. For the 0.4 Mb duplication region on chromosome 21 (OR = 2.23, *P* = 0.0001) the probe-specific results from OncoArray highlighted that the signal is specific to a shorter intergenic region (21:33421860−33459975) between *HUNK* and *LINC00159*.

We observed some evidence of an aggregate directional effect, both for genes and non-genic regions, such that the deletions in aggregate were associated with increased risk. There was also some suggestion that duplications, in aggregate, were associated with a reduced risk. These results suggest that additional associations are present that could be established with a larger dataset. A new GWAS, Confluence (https://dceg.cancer.gov/research/cancer-types/breast-cancer/confluence-project), aims to double the available sample size for breast cancer. This GWAS includes probes specifically designed to assay some of the most significant CNVs observed in this study (those significant at *P* < 0.001), and the sample size should be sufficient to confirm or refute these associations.

## Methods

### Subjects

Data were derived from blood samples from study participants in 66 studies participating in the Breast Cancer Association Consortium (BCAC) and genotyped as part of the OncoArray^9,33^ and iCOGS^8^ collaborations (Supplementary Data 1). Studies included population-based and hospital-based case-control studies, and case-control studies nested within prospective cohorts; we only included data from studies that provided both cases and controls. Phenotype data were based on version 12 of the BCAC database. Cases were diagnosed with either invasive breast cancer or carcinoma-in situ. Oestrogen receptor (ER) status was determined from medical records or tissue microarray evaluation, where available. Analyses were restricted to participants of European ancestry, as defined by ancestry informative principal components^8,9^. Where samples were genotyped on both arrays, we excluded the iCOGS sample as the OncoArray has better genome-wide coverage. After sample quality control (see below), data on 36,980 cases and 34,706 controls with iCOGS genotyping, and 49,808 cases and 41,416 controls with OncoArray genotyping, were available for analysis (Supplementary Data 2).

### Arrays

The Illumina iCOGs genotyping array^8^ includes 211,155 probes for SNVs and short insertions/deletions. Most variants were selected because of previous association in case-control studies for breast prostate and ovarian cancers, or for dense mapping of regions harbouring an association. The OncoArray includes 533,631 probes, of which approximately half were selected from the Illumina HumanCore backbone, a set of SNPs designed to tag the most common variants. The remainder were selected on the basis of evidence of previous association with breast, prostate, ovarian, lung, or colorectal cancer risk. Approximately 32,000 variants on the OncoArray were selected to provide dense coverage of associated loci and known genes. The remainder were mostly selected from lists of common variants ranked by *p*-value, with a small number from lists of candidate variants.

### CNV calling

CNVs were called using the CamCNV pipeline as previously described^16^. In brief, the log R (LRR) intensity measurements and B allele frequency (BAF) for each sample at each probe were exported from Illumina’s Genome Studio software. A principal component adjustment (PCA) was applied to the LRR, grouped by study, to remove noise and batch effects. After removing noisy probes and those in regions with known common CNVs, the LRRs for each probe were converted to z-scores using the mean and standard deviation from all BCAC samples. Circular binary segmentation was applied to the z-scores ordered by probe position for each sample using the DNACopy package^34^. This produces a list of segments for each sample by chromosome where the z-score of consecutive probes changes by more than two standard deviations. Segments with a mean probe z-score between −3.7 and −14 were called as deletions and segments with a mean z-score between +2 and +10 as duplications. We restricted the calls to segments covering a minimum of three and a maximum of 200 probes.

As per the CamCNV pipeline, we then excluded deletions with inconsistent B Allele Frequency and CNVs with a shift in LRR at the sample level that was outside the expected range. The additional CNV exclusions are summarised in Supplementary Data 3. To exclude regions with a high level of noise we also excluded CNVs falling within 1 Mb of telomeres and centromeres and a number of immune loci such as the T-cell receptor genes where somatic mutations in the blood are often observed^35^.

### Sample quality control

Standard sample quality control exclusions were performed, as previously described for the SNP genotype analyses^8,9^. These include exclusions for excess heterozygosity, ancestry outliers, mismatches with other genotyping, and close relatives. A stricter sample call rate of >99% was used for the CNV analysis, compared to >95% used in the genotype analyses. We also excluded any participants for whom a DNA sample was not collected from blood and any that had been whole genome amplified.

In addition, we used two metrics to exclude noisy samples liable to produce an excess of unreliable CNV calls. First, we calculated a derivative log ratio spread (DLRS) figure for each sample as the standard deviation of the differences between LRR for probes ordered by genomic position, divided by the square root of two. This measures the variance in the LRR from each probe to the next averaged over the whole genome and thus is insensitive to large fluctuations such as might be expected between different chromosomes in the same sample. An ideal sample would have a small DLRS as the only variance would come from a small number of genuine CNVs. We calculated the DLRS using the dLRs function in R package ADM3 (https://CRAN.R-project.org/package=ADM3) before and after the PCA. At both stages, we excluded samples with a DLRS more than 3.5 standard deviations above the mean DLRS for that study.

Second, we counted the number of short segments (between three and 200 probes) output by DNACopy for each sample. We observed that the distribution of segment counts was skewed to the right with an excess of samples with a large number of segments. We calculated a cut-off for the maximum number of segments using the following formula where *x* is the segment count for each sample (based on the rationale that the distribution of the true number of segments should be approximately Poisson):

$${y}=2* {{{{{\rm{sqrt}}}}}}({x})$$

$${{{{{\rm{cut}}}}}}-{{{{{\rm{off}}}}}}={{{{{\rm{median}}}}}}({y})+3.5$$

The sample exclusions resulting from these QC steps are summarised in Supplementary Data 4.

### Association tests

All analyses were carried out separately for deletions and duplications, since different types of CNV at the same locus do not necessarily have the same effect on risk. As we were only assessing rare CNVs, we treated all carriers as heterozygotes and did not attempt to identify rare homozygotes.

To account for overlapping CNVs and imprecision in the breakpoints, we assigned individual CNVs to regions. To identify the regions, we moved sequentially along each chromosome, identifying the start as an Oncoarray probe position where deletions were observed in at least five samples, and then the end position as the probe position before the first probe where deletions were observed in fewer than five samples. Regions within five probes of each other were then merged together. The process was repeated for duplications. Regions were also merged such that the major susceptibility genes (*BRCA1, BRCA2,* and *CHEK2*) were included within a single region. We then assigned individual CNVs to regions where at least 90% of the CNV’s length fell within the region. For iCOGS, which generally has less dense probe coverage, we first assigned CNVs to the OncoArray regions where they showed >90% overlap. We then assigned any remaining CNVs to regions defined using the iCOGS probes, using the same procedure. Using this approach, 3306 deletion regions were identified from OncoArray data, 812 of which were also observed using iCOGS data, and 541 regions identified using iCOGS alone. For duplications, there were 2203 OncoArray regions, with 854 also observed using iCOGS data, and 483 iCOGS specific regions.

Associations were evaluated for each region using logistic regression, with breast cancer status as the outcome, and the presence of a CNV in the region (0 or 1) as a covariate to derive a log odds ratio per deletion/duplication. Statistical significance was evaluated using a likelihood ratio test (based on the above model and one excluding CNV as a covariate). The logistic regression analyses were conducted using in-house software (https://ccge.medschl.cam.ac.uk/software/mlogit/). Study and ten ancestry informative principal components, defined separately for each array, were also included as covariates. The analyses were conducted separately for the iCOGS and OncoArray and then combined in a standard fixed effect meta-analysis using the METAL software (after first deriving the standard error of the log-odds ratio from the likelihood test statistic)^36^. To avoid regions with too few observations, we excluded regions with fewer than 24 deletions or duplications (~0.015% of samples). Associations significant at *P* < 0.001 were considered noteworthy.

To detect more precisely the location of association signals, we also generated results for each probe. We created a vector of pseudo-genotypes for each probe with samples, such that a deletion covering that probe was coded as 1 and all other samples were coded as 0. We generated a similar set of genotypes for duplications. The results were analysed using logistic regression, as above.

To test for association between CNVs affecting the coding sequence of genes, in aggregate, and breast cancer risk, we identified samples with a deletion or duplication overlapping the exons of each gene. Exon positions were downloaded from the UCSC Genome Browser hg19 knownGene table. We used logistic regression to generate a log odds ratio (OR) for carriers of coding variants covering each gene, adjusted for study, as above. We generated results for each array and then for carriers combined across both arrays. For the combined analyses we treated studies with samples on both arrays as separate studies.

To calculate BFDPs we assumed a log-normally distributed prior effect size as described by Wakefield^24^. The prior log(OR) was determined by assuming a 95% probability that the OR was less than some bound *K*, where *K* = 3 for the regional and gene-based analysis, except for *BRCA1* and *BRCA2* where *K* = 20 was assumed. The prior probability of association was assumed to be 0.001 for the regional analysis, 0.99 for *BRCA1, BRCA2, ATM,* and *CHEK2* and 0.002 for other genes. For the gene-based analysis, only positive associations were considered as the prior evidence for all genes was in favour of PTVs being positively associated with risk.

To determine whether there was a tendency for CNVs to be associated with an excess, or deficit, of risk across genes or regions, we computed signed z-scores as the square root of the chi-squared statistic for each gene, multiplied by ±1 depending on whether the effect estimate was positive or negative. These were ranked and normalised summed z-scores, based on the r most significant associations, were derived. The overall test statistic was the maximum summed z-score over all possible values of *r*:

1 $$U=\begin{array}{c}{\max }\\ r\le n\end{array}\frac{1}{\sqrt{r}}\mathop{\sum }\limits_{j=1}^{r}{z}_{j}$$

Where *n* is the total number of genes/regions being tested. The significance of *U* was then determined by permutation, randomly permuting case-control labels within study 50 times.

### Ethical approval

All participating studies were approved by their appropriate ethics review board and all subjects provided written informed consent.

### Reporting summary

Further information on research design is available in the Nature Research Reporting Summary linked to this article.

## Supplementary information

Supplementary Information

Description of Additional Supplementary Files

Supplementary Data 1-21

Reporting Summary

## Data Availability

Full summary statistics for the regions and probes analysed are available in the Supplementary Data. This includes the source data used to produce Figs. 2 and 3. The majority of the OncoArray dataset analysed in this study is available in the dbGap repository, Study ID: phs001265.v1.p1 (https://www.ncbi.nlm.nih.gov/projects/gap/cgi-bin/study.cgi?study_id=phs001265.v1.p1). The iCOGS dataset and complete OncoArray dataset cannot be made publicly available due to restraints imposed by the ethics committees of individual studies; requests for data can be made to the corresponding author or the Data Access Coordination Committee (DACC) of BCAC (http://bcac.ccge.medschl.cam.ac.uk/).

## Code availability

Code for the CamCNV calling algorithm and a test dataset with OncoArray genotyping is available at the project home page (https://github.com/jgd29/CamCNV).
