# Supplementary figures and images for: Rare germline copy number variants (CNVs) and breast cancer risk
Source: Commun Biol. 2022 Jan 18;5:65. doi: 10.1038/s42003-021-02990-6 (PMC8766486; doi:10.1038/s42003-021-02990-6)

**Supplementary Figure 1. Overlap of called CNVs with 1000 Genomes Variants**

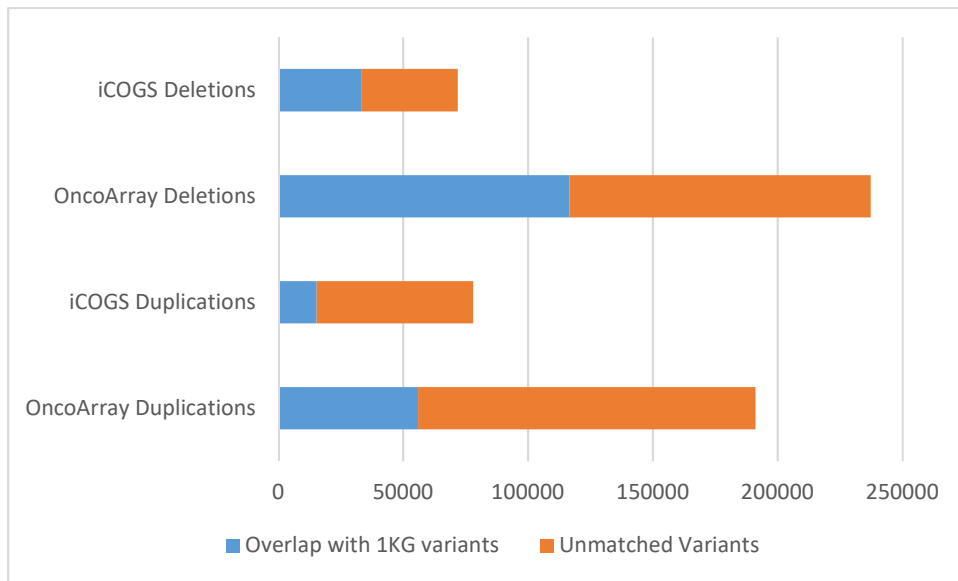

Supplement: Supplementary file 1 — Supplementary Information [file 42003_2021_2990_MOESM1_ESM.pdf]
