# Supplementary material for: Rare germline copy number variants (CNVs) and breast cancer risk
Source: Commun Biol. 2022 Jan 18;5:65. doi: 10.1038/s42003-021-02990-6 (PMC8766486; doi:10.1038/s42003-021-02990-6)
Supplement: Supplementary file 2 — Description of Additional Supplementary Files [file 42003_2021_2990_MOESM2_ESM.pdf]

Description of Additional Supplementary Files

File name: Supplementary Data 1-21

Description:

Supplementary Data 1: BCAC Studies

Supplementary Data 2: Sample Numbers

Supplementary Data 3: CNV QC Exclusions

Supplementary Data 4: Sample QC Exclusions

Supplementary Data 5: Summary of CNVs passing QC

Supplementary Data 6: Observed CNV fequency at array probes

Supplementary Data 7: Overall risk for deletion regions

Supplementary Data 8: Overall risk for duplication regions

Supplementary Data 9: Oncoarray deletion probe results

Supplementary Data 10: iCOGs deletion probe results

Supplementary Data 11: Oncoarray duplication probe results

Supplementary Data 12: iCOGs duplication probe results

Supplementary Data 13: Results by subtype for deletion regions

Supplementary Data 14: Results by subtype for duplication regions

Supplementary Data 15: Conditional analyses with known SNPs

Supplementary Data 16: Gene burden for deletions

Supplementary Data 17: Gene burden for duplications

Supplementary Data 18: Gene burden for deletions by subtype

Supplementary Data 19: Gene burden for duplications by subtype

Supplementary Data 20: Bayesian False Discovery Probabilities (BFDPs) for regional results

Supplementary Data 21: Bayesian False Discovery Probabilities (BFDPs) for gene burden results
