# Supplementary material for: Rare germline copy number variants (CNVs) and breast cancer risk
Source: Commun Biol. 2022 Jan 18;5:65. doi: 10.1038/s42003-021-02990-6 (PMC8766486; doi:10.1038/s42003-021-02990-6)
Supplement: Supplementary file 4 — Reporting Summary [file 42003_2021_2990_MOESM4_ESM.pdf]

nature portfolio

Corresponding author(s): Joe Dennis

Last updated by author(s): Jun 18, 2021

Reporting Summary

Nature Portfolio wishes to improve the reproducibility of the work that we publish. This form provides structure for consistency and transparency in reporting. For further information on Nature Portfolio policies, see our Editorial Policies and the Editorial Policy Checklist.

Statistics

For all statistical analyses, confirm that the following items are present in the figure legend, table legend, main text, or Methods section.

n/a	Confirmed	
☐	☒	The exact sample size (*n*) for each experimental group/condition, given as a discrete number and unit of measurement
☒	☐	A statement on whether measurements were taken from distinct samples or whether the same sample was measured repeatedly
☐	☒	The statistical test(s) used AND whether they are one- or two-sided<br>*Only common tests should be described solely by name; describe more complex techniques in the Methods section.*
☐	☒	A description of all covariates tested
☒	☐	A description of any assumptions or corrections, such as tests of normality and adjustment for multiple comparisons
☒	☐	A full description of the statistical parameters including central tendency (e.g. means) or other basic estimates (e.g. regression coefficient) AND variation (e.g. standard deviation) or associated estimates of uncertainty (e.g. confidence intervals)
☐	☒	For null hypothesis testing, the test statistic (e.g. *F*, *t*, *r*) with confidence intervals, effect sizes, degrees of freedom and *P* value noted<br>*Give P values as exact values whenever suitable.*
☐	☒	For Bayesian analysis, information on the choice of priors and Markov chain Monte Carlo settings
☒	☐	For hierarchical and complex designs, identification of the appropriate level for tests and full reporting of outcomes
☒	☐	Estimates of effect sizes (e.g. Cohen's *d*, Pearson's *r*), indicating how they were calculated

Our web collection on statistics for biologists contains articles on many of the points above.

Software and code

Policy information about availability of computer code

Data collection	Illumina Genome Studio, R version 3.6.3 (bigPCA, DNACopy, ADM3 packages), example CNV calling code for CamCNV pipeline at https://github.com/jgd29/CamCNV
Data analysis	Logistic regression with in-house package (https://ccge.medschl.cam.ac.uk/software/mlogit/), METAL meta analysis (https://genome.sph.umich.edu/wiki/METAL) Latest stable METAL from generic-metal-2011-03-25.

For manuscripts utilizing custom algorithms or software that are central to the research but not yet described in published literature, software must be made available to editors and reviewers. We strongly encourage code deposition in a community repository (e.g. GitHub). See the Nature Portfolio guidelines for submitting code & software for further information.

Data

Policy information about availability of data

All manuscripts must include a data availability statement. This statement should provide the following information, where applicable:

- Accession codes, unique identifiers, or web links for publicly available datasets
- A description of any restrictions on data availability
- For clinical datasets or third party data, please ensure that the statement adheres to our policy

Full summary statistics for the regions and probes analysed are available in the Supplementary Tables. The majority of the OncoArray dataset analyzed in this study is available in the dbGap repository, Study ID: phs001265.v1.p1 (https://www.ncbi.nlm.nih.gov/projects/gap/cgi-bin/study.cgi?study_id=phs001265.v1.p1). The iCOGS dataset and complete OncoArray dataset cannot be made publicly available due to restraints imposed by the ethics committees of individual studies; requests for data can be made to the corresponding author or the Data Access Coordination Committee (DACC) of BCAC (http://bcac.ccge.medschl.cam.ac.uk/).

March 2021

Field-specific reporting

Please select the one below that is the best fit for your research. If you are not sure, read the appropriate sections before making your selection.

☒ Life sciences ☐ Behavioural & social sciences ☐ Ecological, evolutionary & environmental sciences

For a reference copy of the document with all sections, see nature.com/documents/nr-reporting-summary-flat.pdf

Life sciences study design

All studies must disclose on these points even when the disclosure is negative.

Sample size	*Describe how sample size was determined, detailing any statistical methods used to predetermine sample size OR if no sample-size calculation was performed, describe how sample sizes were chosen and provide a rationale for why these sample sizes are sufficient.*
Data exclusions	Standard genotyping QC metrics to exclude poor quality samples (heterozygosity, call rate > 99%). Also excluded any samples not from blood and used DLRS and segment count metrics to exclude samples with excessive noise. Metrics and rationale for exclusion of probes, loci and CNV calls as described in CamCNV methods paper (PMID:33020983).
Replication	Meta analysis results are from two separate arrays although, as described, probes were not always available to detect same CNV regions on both arrays.
Randomization	NA
Blinding	NA

Reporting for specific materials, systems and methods

We require information from authors about some types of materials, experimental systems and methods used in many studies. Here, indicate whether each material, system or method listed is relevant to your study. If you are not sure if a list item applies to your research, read the appropriate section before selecting a response.

Materials & experimental systems

n/a	Involved in the study	
☒	☐	Antibodies
☒	☐	Eukaryotic cell lines
☒	☐	Palaeontology and archaeology
☒	☐	Animals and other organisms
☐	☒	Human research participants
☒	☐	Clinical data
☒	☐	Dual use research of concern

Methods

n/a	Involved in the study	
☒	☐	ChIP-seq
☒	☐	Flow cytometry
☒	☐	MRI-based neuroimaging

Human research participants

Policy information about studies involving human research participants

Population characteristics	Women of European ancestry as defined by genotypes.
Recruitment	A variety of recruitment methods were used by the BCAC studies listed in ST1 - full details available on request.
Ethics oversight	Each BCAC study has their own ethical oversight board - full details available on request.

Note that full information on the approval of the study protocol must also be provided in the manuscript.
